# Dr. Joseph G. Nancrede

**Published:** 1857-05

**Authors:** 


Dr. Joseph Gerrard Nancrede.—By the recent death of this gentleman, a void has been made in the ranks of the medical profession in Philadelphia. One of its most intelligent and best educated members,—he gave himself up with zeal, assiduity, and success to the discharge of its various and trying duties, for the long period of nearly forty-four years. During this time he bore himself well and honorably, without show or boast, winning and retaining the respect of his fellow-practitioners, and the confidence and love of a large circle of patients of all classes, who found in him a union of kindness with skill, a sympathizing friend and a discriminating physician.

Doctor Nancrede was a native of Boston. His father was a Frenchman, an officer under Rochambeau, and a friend of Lafayette in the war of our independence, for his gallantry and good conduct in which, he was, we are told, honored with a favorable notice by Washington himself. The family could boast of its quarterings of nobility, by its descent from a Gerrard, an Englishman, who entered the French army in the reign of Charles VII, and afterwards, by the acquisition of an estate, took the name of

Nancrede. The subject of our present notice, Joseph G., was sent, in his boyhood, to a Catholic seminary, at Montreal, where he formed an intimacy with young Papineau, who afterwards played so conspicuous a part in Canadian politics, and with whom he kept up a friendly intercourse through life. Thence he went to Paris, in which city he received his collegiate education, and prosecuted his medical studies. His father, at this time, resided in St. Germain.

On his return to his native country, young Nancrede attended the lectures in the medical department of the University of Pennsylvania, and obtained the honors of the doctorate in the year 1813. Thus qualified, he began to practise his profession in Louisville, Kentucky, but soon returned to Philadelphia, where he spent the remainder of his life. In 1822 Doctor Nancrede married a daughter of Commodore Truxton, whose demise preceded his own by eight years. In the death of this estimable and intelligent lady, her husband received a shock, from the effects of which he never recovered. In fact, we may trace to this event, and its subsequent depressing influence, the diseases under which Dr. Nancrede himself sank. The more obvious malady, phthisis pulmonalis, was associated with a less clearly determined, though still evident disorder of the circulatory apparatus, which, for some months before his death, prevented him from taking exercise on foot, or even walking a short distance without a feeling of exhaustion, and labored respiration. He was scarcely troubled with cough, had little expectoration, and no hectic fever, or night sweats.

Doctor Nancrede, notwithstanding his fitness, by suitable attainments and experience, and a good style of writing, did not often appear as an author. At a very early date, he was associated with his elder brother, Dr. Nicholas C. Nancrede, in bringing out a translation of Legallois' Experiments on the Principles of Life, &c. He afterwards made a translation and abridgment of Orfila's work on Toxicology. He wrote occasional papers for the medical journals: of these, one was on Mania a Potu, in the first volume of the Medical Recorder; another was, An Account of the Doctrine of Fevers, by Broussais, in the eighth volume of Chapman's Philadelphia Journal. In the fourteenth volume of this work appeared his Memoir of Dr. Mongez; and in the sixteenth volume of the American Journal of Medical Sciences, Observations on a Case of Cæsarean Operation, occurring in his own practice, in which both mother and child were preserved. He was instrumental in procuring the first use to be made here of Monœsia. He was also active in causing trials to be made of the sphygmometer, and translated an account of its use and application.

Dr. Nancrede died on the 2d of February, 1857, in the 64th year of his age. He died as he had lived, in the communion of the Roman Catholic Church. Among the pall-bearers at his funeral, was his old friend Mr. Papineau, who, with his family was spending the winter in Philadelphia. Dr. Nancrede, in default of issue, left his adopted son, Dr. Sam. J. G. Nancrede, the heir to his estate. This gentleman is the nephew of Mrs. Nancrede.

